# Supplementary material for: Direct measurement of TRPV4 and PIEZO1 activity reveals multiple mechanotransduction pathways in chondrocytes
Source: eLife. 2017 Jan 30;6:e21074. doi: 10.7554/eLife.21074 (PMC5279942; doi:10.7554/eLife.21074)
Supplement: Figure 2—source data 1. — Chondrocytes were isolated from C57Bl/6 mice. For each sample (chondrocyte phenotype and dedifferentiated phenotype) the number of litters, recorded cells and number of cells that respond to pillar deflections are shown along with the total number of stimulation points (corresponding to the number of distinct pili deflected) and the total number of measurements (i.e. individual deflections). For each recorded current, the latency and the current amplitude were measured, and the activation time constant and current decay were obtained from a mono-exponential fit of the data. The mean ± s.e.m. and the median are displayed for each kinetic parameter. DOI: http://dx.doi.org/10.7554/eLife.21074.006 [file elife-21074-fig2-data1.docx]

|  | **WT C57Bl/6** | |
| --- | --- | --- |
|  | **Chondrocyte** | **Dedifferentiated** |
| Number of litters | 5 | 5 |
| Cells | 27 | 17 |
| Responding cells | 24 | 15 |
| Stimulation points | 32 | 22 |
| Measurements | 399 | 214 |
| No. of currents | 99 | 109 |
| Current amplitude (pA) mean (± s.e.m.)  Median | 188.6 ± 24.4  97.3 | 202.8 ± 29.2  97.6 |
| Latency (ms) mean (± s.e.m.)  Median | 3.6 ± 0.3  2.6 | 3.1 ± 0.3  1.6 |
| τ1 (ms) mean (± s.e.m.)  Median | 1.7 ± 0.3  0.7 | 1.4 ± 0.3  0.5 |
| τ2 (ms) mean (± s.e.m.)  Median | 47.7 ± 8.6  16.7 | 135.4 ± 31.8  28.7 |

Source Data FIgure 2
