# Supplementary material for: Direct measurement of TRPV4 and PIEZO1 activity reveals multiple mechanotransduction pathways in chondrocytes
Source: eLife. 2017 Jan 30;6:e21074. doi: 10.7554/eLife.21074 (PMC5279942; doi:10.7554/eLife.21074)
Supplement: Figure 3—source data 1. — (A) Statistical comparison of deflection-gated mechanoelectrical transduction responses. For each individual cell, currents were binned in the indicated size ranges (in nm) and the current amplitudes within each bin averaged and then averaged across cells. Bins were subsequently tested for normal distribution and subsequently compared with a Student’s t-test (parametric data sets) or a Mann Whitney test (non-parametrical data). The p values are shown for significant comparisons, ‘NS’ indicates no significant differences and ‘NA’ is shown when all measurements within a bin were equal to zero. The number of compared points is shown in brackets. An ordinary two-way ANOVA was used to compare the cellular response over the range of stimuli, reported are the p value and F statistic (including DFn, DFd). (B) Statistical comparison of stretch-gated mechanoelectrical transduction in chondrocytes. Chondrocytes were isolated from WT mice, expanded and encapsulated in alginate. After deposition on coverslips for measurement, cells were analyzed using HSPC. For each condition, the number of litters, recorded membrane patches and maximal current (pA) are shown. Data are displayed as mean ± s.e.m. Conditions were compared with Student’s t-test (parametric data sets), and the p values are shown for significant comparisons, ‘NS’ indicates no significant. DOI: http://dx.doi.org/10.7554/eLife.21074.008 [file elife-21074-fig3-data1.docx]

| **Stimulus-response curves: Statistics** | | | | | | | |
| --- | --- | --- | --- | --- | --- | --- | --- |
|  | 0-10 | 10-50 | 50-100 | 100-250 | 250-500 | 500-1000 | Ordinary Two-way ANOVA |
| **WT C57Bl/6** | | | | | | |  |
| Chondrocytes vs de-differentiated | NA | * *P*=0.02  (17,12) | NS  (19,12) | ** *P*=0.004  (22,13) | NS  (19,10) | NS  (15,7) | * *P*=0.03  F (1, 146) = 4.631 |

| **HSPC outside out patches** | | |
| --- | --- | --- |
|  | WT chondrocytes | WT dedifferentiated cells |
| Number of litters | 3 | 3 |
| Cell patches | 12 | 13 |
| Maximal current (pA) mean (± s.e.m.) | 45.2 ± 7.5 | 48.6 ± 6.7  vs WT chondrocytes NS |

**Source data figure 3**
