# Supplementary material for: Direct measurement of TRPV4 and PIEZO1 activity reveals multiple mechanotransduction pathways in chondrocytes
Source: eLife. 2017 Jan 30;6:e21074. doi: 10.7554/eLife.21074 (PMC5279942; doi:10.7554/eLife.21074)
Supplement: Figure 4—source data 1. — (A) Electrophysiological characteristics of WT, Trpv4-/- and miRNA-treated chondrocytes. Chondrocytes were isolated from C57Bl/6 and Trpv4-/- mice, expanded, transfected (in the case of Scrambled and Piezo1 miRNA constructs) and encapsulated in alginate. For each condition, the number of litters, recorded cells and number of cells that respond to pillar deflections are shown. The total number of stimulation points (corresponding to the number of distinct pili deflected) and the total number of measurements (i.e. individual deflections) are displayed. For each recorded current, the latency and the current amplitude were measured, and the activation time constant and current decay were obtained from a mono-exponential fit of the data. The mean ± s.e.m. and the median are displayed for each kinetic parameter. (B) Statistical comparison of deflection-gated mechanoelectrical transduction responses. For each individual cell, currents were binned in the indicated size ranges (in nm) and the current amplitudes within each bin averaged and then averaged across cells. Bins were tested for normal distribution and subsequently compared with a Student’s t-test (parametric data sets) or a Mann-Whitney test (non-parametrical data). The p values are shown for significant comparisons, ‘NS’ indicates no significant differences and ‘NA’ is shown when all measurements within a bin were equal to zero or data were not enough to perform the comparison. The number of compared points is shown in brackets. An ordinary two-way ANOVA was used to compare the cellular response over the range of stimuli, reported are the p value and F statistic (including DFn, DFd). DOI: http://dx.doi.org/10.7554/eLife.21074.010 [file elife-21074-fig4-data1.docx]

|  | **WT C57Bl/6** | ***Trpv4^-/-^*** | **WT C57Bl/6** | | ***Trpv4^-/-^*** |
| --- | --- | --- | --- | --- | --- |
|  |  |  | **Scrambled miRNA** | ***Piezo1*-targeting miRNA** | ***Piezo1*-targeting miRNA** |
| Number of litters | 5 | 4 | 2 | 3 | 3 |
| Cells | 27 | 13 | 22 | 12 | 11 |
| Responding cells | 24 | 6 | 19 | 6 | 2 |
| Stimulation points | 32 | 19 | 28 | 18 | 17 |
| Measurements | 399 | 175 | 352 | 208 | 227 |
| No. of currents | 99 | 12 | 64 | 15 | 5 |
| Latency (ms) mean (± s.e.m.)  Median | 3.6 ± 0.3  2.6 | 7.8 ± 1.6  8.3 | 4.3 ± 0.7  3.9 | 8.11 ± 1.9  4.6 | 6.7 ± 2.3  4.6 |
| τ1 (ms) mean (± s.e.m.)  Median | 1.7 ± 0.3  0.7 | 0.32 ± 0.06  0.26 | 1.3 ± 0.2  0.75 | 1.1 ± 0.3  0.9 | 1.3 ± 0.5  0.7 |
| τ2 (ms) mean (± s.e.m.)  Median | 47.7 ± 8.6  16.7 | 45.1 ± 24.7  17.7 | 67.8 ± 13.5  22.4 | 26.3 ± 16.6  2.2 | 9.3 ± 4.2  5.8 |

| **Stimulus-response curves: Statistics** | | | | | | | |
| --- | --- | --- | --- | --- | --- | --- | --- |
|  | 0-10 | 10-50 | 50-100 | 100-250 | 250-500 | 500-1000 | Ordinary Two-way ANOVA |
| **WT-scrambled miRNA vs *Piezo1* miRNA (B57Bl/6)** | | | | | | |  |
| Chondrocytes vs chondrocytes | NA | NS  (21,11) | NS  (21,11) | NS  (22,11) | NS  (14,10) | NS  (7,7) | NS |
| **WT vs *Trpv4^-/-^* (C57Bl/6)** | | | | | | |  |
| Chondrocytes vs chondrocytes | NA | NS  (20,11) | NS  (21,10) | NS  (25,12) | NS  (22,11) | NS  (16,6) | * *P*=0.04  F (1, 148) = 4.035 |
| **WT-scrambled miRNA vs *Trpv4^-/-^*-*Piezo1* miRNA** | | | | | | |  |
| Chondrocytes vs chondrocytes | NA | NS  (21,10) | NA  (21,10) | NS  (22,10) | NA  (14,8) | NA  (7,7) | * *P*=0.04  F (1, 128) =  3.984 |

Source Data Figure 4
