# Supplementary material for: Direct measurement of TRPV4 and PIEZO1 activity reveals multiple mechanotransduction pathways in chondrocytes
Source: eLife. 2017 Jan 30;6:e21074. doi: 10.7554/eLife.21074 (PMC5279942; doi:10.7554/eLife.21074)
Supplement: Figure 6—source data 1. — Chondrocytes were isolated from WT and Trpv4-/- mice, expanded, transfected (in the case of Scrambled and Piezo1 miRNA constructs) and encapsulated in alginate. For each condition, the number of litters, recorded membrane patches and maximal current (pA) are shown. Data are displayed as mean ± s.e.m. Conditions were compared with Student’s t-test (parametric data sets) and the p values are shown for significant comparisons, ‘NS’ indicates no significant. DOI: http://dx.doi.org/10.7554/eLife.21074.014 [file elife-21074-fig6-data1.docx]

|  | WT chondrocytes | Scrambled-miRNA chondrocytes | *Piezo1*-miRNA chondrocytes | *Trpv4^-/-^* chondrocytes | *Piezo1*-miRNA in *Trpv4^-/-^* chondrocytes |
| --- | --- | --- | --- | --- | --- |
| Number of litters | 3 | 2 | 2 | 3 | 2 |
| Cell patches | 12 | 11 | 10 | 7 | 7 |
| Maximal current (pA) mean (± s.e.m.) | 45.2 ± 7.5 | 72.8 ± 14.3  vs WT chondrocytes NS | 4.1 ± 0.8  vs Scrambled miRNA chondrocytes *** *P*=0.0002 | 51.4 ± 12.9  vs WT chondrocytes NS | 5.2 ± 0.9  vs Scrambled miRNA chondrocytes ** *P*=0.002  vs *Piezo1* miRNA chondrocytes NS |

Source Data Figure 6
