# Supplementary material for: Direct measurement of TRPV4 and PIEZO1 activity reveals multiple mechanotransduction pathways in chondrocytes
Source: eLife. 2017 Jan 30;6:e21074. doi: 10.7554/eLife.21074 (PMC5279942; doi:10.7554/eLife.21074)
Supplement: Figure 8—source data 1. — (A) Electrophysiological characteristics of HEK-293 cells overexpressing either TRPV4 or PIEZO1. HEK-293 cells were transiently transfected with a plasmid encoding either TRPV4 or PIEZO1. For each condition the number of transfections, total number of recorded cells and number of stimuli are indicated. For each recorded current, the latency and the current amplitude were measured, and the activation time constant and current decay were obtained from a mono-exponential fit. Every kinetic parameter is shown as mean ± s.e.m. (B) Statistical comparison of deflection-gated mechanoelectrical transduction responses. For each individual cell, currents were binned in the indicated size ranges (in nm) and the current amplitudes within each bin averaged and then averaged across cells. Bins were subsequently tested for normal distribution and subsequently compared with a Student’s t-test (parametric data sets) or a Mann Whitney test (non-parametrical data). The p values are shown for significant comparisons, ‘NS’ indicates no significant differences. The number of compared points is shown in brackets. An ordinary two-way ANOVA was used to compare the cellular response over the range of stimuli, reported are the p value and F statistic (including DFn, DFd). DOI: http://dx.doi.org/10.7554/eLife.21074.021 [file elife-21074-fig8-data1.docx]

| **Deflection-gated currents** |
| --- |

|  | HEK-293 TRPV4 | HEK-293 PIEZO1 |
| --- | --- | --- |
| Transfections  Cells  Measurements | 3  8  109 | 4  12  154 |
| Latency (mean ± s.e.m.) (ms) | 1.5 ± 0.2 | 1.5 ± 0.2 |
| τ_1_ (mean ± s.e.m.) (ms) | 0.48 ± 0.05  * *P*=0.04 (33,39) | 1.2 ± 0.25 |
| τ_2_ (mean ± s.e.m.) (ms) | 16 ± 7  ** *P*=0.005 (29, 26) | 76 ± 31 |

| **Stimulus-response curve: Statistics** | | | | | | | |
| --- | --- | --- | --- | --- | --- | --- | --- |
|  | 0-10 | 10-50 | 50-100 | 100-250 | 250-500 | 500-1000 | Ordinary Two-way ANOVA |
| PIEZO1 vs TRPV4 overexpression | NS  (8,5) | * *P*=0.01  (12,8) | * *P*=0.02  (10,6) | * *P*=0.03  (12,7) | ** *P*=0.001  (12,4) |  | **** *P*<0.0001  F (1, 86) = 19.96 |

Source data figure 8
